# Association Between Sleep Apnea and Dry Eye Disease in the All-of-Us Program

**DOI:** 10.3390/biomedicines14010024

**Published:** 2025-12-22

**Authors:** Annie Zhang, Jocelyn He, Gui-Shuang Ying

**Affiliations:** 1 Drexel University College of Medicine, Philadelphia, PA 19104, USA; az599@drexel.edu; 2 Center for Preventive Ophthalmology and Biostatistics, Department of Ophthalmology, Scheie Eye Institute, Perelman School of Medicine at the University of Pennsylvania, Philadelphia, PA 19104, USA; jocelyn.he@pennmedicine.upenn.edu

**Keywords:** obstructive sleep apnea, dry eye disease, All-of-Us, cross-sectional, epidemiology

## Abstract

**Purpose:** The aim of this study was to investigate the association between obstructive sleep apnea (OSA) and the prevalence of dry eye disease (DED) and meibomian gland dysfunction (MGD) using the All-of-Us Research Program (AoURP) dataset from a large, demographically diverse U.S. population. **Methods:** In this cross-sectional, matched case–control study, participants with documented OSA were exactly matched 1:3 by age, gender, race, and ethnicity to controls without OSA. Associations between OSA and DED and MGD were evaluated using univariate and multivariate logistic regression models adjusted for obesity, diabetes, smoking, hypertension, hyperlipidemia, hypothyroidism, and cardiovascular disease at the time of enrollment. **Results:** Among the 628,649 AoURP participants, 59,804 individuals had OSA and 179,412 matched controls were identified with the same demographics (mean age 61.95 years; 54.0% female; 12.5% Hispanic; 62.3% non-Hispanic White; 15.5% non-Hispanic Black). Compared to controls, OSA participants had significantly higher rates of smoking (13.7% vs. 10.9%), obesity (68.4% vs. 13.2%), diabetes (43.3% vs. 11.7%), hypertension (76.4% vs. 28.2%), hyperlipidemia (74.5% vs. 27.5%), hypothyroidism (24.7% vs. 8.1%), and cardiovascular disease (43.1% vs. 12.8%) (all *p* < 0.001). Compared to matched controls, the prevalence of DED was significantly higher in the OSA group (19.4% vs. 5.8%), with an adjusted odds ratio (OR) of 1.76 (95% confidence interval (95% CI), 1.70–1.82; *p* < 0.001). MGD prevalence was also higher in the OSA group (2.6% vs. 1.0%), with an adjusted OR of 1.43 (95% CI, 1.32–1.55; *p* < 0.001). **Conclusions:** In this large, demographically diverse U.S. population, obstructive sleep apnea was independently associated with a higher prevalence of both dry eye disease and meibomian gland dysfunction. These findings provide large-scale U.S. evidence and suggest that screening for ocular surface disease may be warranted in patients with OSA to improve detection and management.

## 1. Introduction

Obstructive sleep apnea (OSA) is a highly prevalent, yet underdiagnosed, disorder affecting an estimated 9–38% of the general adult population worldwide, with increasing prevalence in older adults and those with comorbidities such as obesity and hypertension [1]. OSA is characterized by repeated episodes of upper airway collapse during sleep resulting in intermittent hypoxia, hypercapnia, surges of sympathetic activity and sleep fragmentation [2]. The deleterious effects of OSA extend well beyond disturbed sleep and contribute to increased risks of hypertension, coronary artery disease, stroke, metabolic syndrome, and impaired neurocognitive function [3,4].

Recent evidence suggests that OSA may also contribute to ocular surface disorders, particularly dry eye disease (DED) [5,6]. DED is a chronic, multifactorial disorder of the ocular surface characterized by tear film instability and hyperosmolarity, resulting in ocular discomfort, visual disturbance, and inflammation [7]. In the United States, DED affects an estimated 6.8% of the adult population and is associated with significant impairments in quality of life and daily functioning [8]. The current proposed biological mechanisms linking OSA and DED include chronic intermittent hypoxia-induced oxidative stress, systemic inflammation, and sympathetic overactivation, which may disrupt lacrimal gland function, alter tear film composition, and exacerbate meibomian gland dysfunction [9,10]. Meibomian gland dysfunction (MGD) is a chronic, diffuse abnormality of the meibomian glands, characterized by terminal duct obstruction and/or changes in glandular secretion. MGD has emerged as the leading cause of evaporative DED, affecting approximately 35.8% of the global population [11]. MGD involves chronic abnormalities in meibomian gland structure and function, resulting in altered meibum secretion that destabilizes the tear film and causes ocular surface inflammation [11].

While preliminary studies have reported associations between OSA and DED, large population-based studies conducted in the United States remain limited and show conflicting findings [12,13]. Previous research has been limited by small sample sizes, lack of diversity, and insufficient control for comorbidities and confounders. For instance, a meta-analysis of 1526 subjects demonstrated that individuals with OSA exhibited significantly poorer tear breakup time, Schirmer test results, and Ocular Surface Disease Index scores compared to controls without OSA, whereas other investigations found no independent association between OSA and DED after adjusting for confounders such as age and diabetes [12,14]. Additionally, much of the existing literature derives from homogenous Asian cohorts, where population-specific factors may limit generalizability to diverse populations such as the United States [10,12,15]. These inconsistencies highlight the ongoing uncertainty regarding whether the association between OSA and DED reflects a direct causal relationship or arises from shared risk factors.

The All-of-Us Research Program (AoURP), launched by the National Institutes of Health in 2018, aims to enroll over one million participants across the United States, capturing a broad spectrum of demographic, genetic, environmental, and health data, including electronic health records (EHR), survey data, and biospecimens [16]. By leveraging the AoURP, this study will provide a nationally representative validation of these relationships, offering translational insights into population-level epidemiology across diverse and historically underrepresented groups in the U.S.

This study leverages the scale and diversity of the AoURP to address the existing research gap. Specifically, the aim of this study is to investigate whether the association between OSA and both DED and MGD is independent of common comorbidities in a large, demographically diverse U.S. population. We hypothesized that OSA is independently associated with a higher prevalence of DED and MGD.

## 2. Materials and Methods

This study utilized data from the All of Us Research Program (AoURP), which emphasizes the enrollment of diverse and historically underrepresented populations [16]. Officially established in May 2018, the program aims to recruit participants from various backgrounds across the United States. As of 24 May 2025, more than 746,000 individuals had been enrolled in the program, with 710 sites collecting biospecimens and measurements, and over 470,000 electronic health records (EHRs) included in the AoURP database [17]. Participants can join through affiliated healthcare organizations or self-enroll directly via the AoURP website, and the written informed consent for research participation was obtained from all participants at the time of enrollment [16]. Data include EHRs, survey responses, and genetic information, enabling researchers to conduct high-powered, generalizable, and equity-focused studies. In the EHR data, the health conditions were identified using the Systematized Nomenclature of Medicine–Clinical Terms (SNOMED-CT). This study was exempt from Institutional Review Board (IRB) ethical approval for human subject research because only deidentified data were analyzed from the AoURP. Access to participant-level data in All of Us requires a data passport, granted through a six-step process that includes institutional affiliation, account creation, identity verification, ethics training, and agreeing to the data use code of conduct.

### 2.1. Study Design and Population

This study used the cross-sectional, matched case–control design to evaluate the association between OSA and both DED and MGD. The cases were participants with a documented OSA diagnosis (SNOMED code: 78275009). The controls were individuals without OSA. Cases and controls were 1:3 exactly matched by age, sex, race, and ethnicity (i.e., one OSA case was matched with 3 controls who had the same age, sex, race and ethnicity as the case). A 1:3 matching ratio was chosen to optimize statistical power while maintaining balance in demographics. The DED status and MGD, the outcome measures of the study, was identified by SNOMED codes (DED: 162290004, 46152009, 397549002, 16237381000119100, 16237461000119104, 16237421000119109; MGD: 397549002).

To control the confounders, the status of other diseases at the time of enrollment was identified including: obesity (SNOMED code: 238136002), diabetes mellitus (SNOMED code: 73211009), hypertension (SNOMED code: 38341003), hyperlipidemia (SNOMED code: 55822004), hypothyroidism (SNOMED code: 40930008), and cardiovascular disease (SNOMED code: 64715009).

### 2.2. Statistical Analysis

Descriptive analyses were performed for OSA group and control group using mean, standard deviation (SD) for continuous measure, count and percentage for categorical measures. Two sample *t*-test was used for comparison of means, and Chi-squared test was used to compare proportions. Univariate and multivariate logistic regression models on the complete data were used to evaluate whether OSA is associated with DED and MGD. Multivariate logistic regression model was adjusted by current smoking status, obesity, diabetes, hypertension, hyperlipidemia, hypothyroidism, and cardiovascular disease determined at the enrollment. Odds ratios (OR), adjusted OR, and their 95% confidence intervals (95% CI) were calculated from univariate and multivariate logistic regression models respectively. All statistical analyses were performed using data of Version 8 (as of February 2025) in the All-of-Us workstation using R version 4.3.1. Matching was performed in R using the MatchIt package with a 1:3 ratio was selected to increase statistical precision while maintaining covariate balance. Two-sided *p* values < 0.05 was considered statistically significant.

## 3. Results

### 3.1. Characteristics of Participants

Among the 628,649 participants of AoURP, 59,804 participants with OSA and 179,412 matched controls without OSA were selected for evaluating association between OSA and DED. The demographic characteristics and comorbidities in OSA group and matched control group are reported in Table 1.

Through matching demographics, both groups had same mean age of 62.0 years (SD = 13.6 years), 54.0% female, 12.5% Hispanic or Latino, 62.3% non-Hispanic White, 15.5% non-Hispanic Black (15.5%). The OSA group had significantly higher proportions of current smoking (13.7% vs. 10.9%, *p* < 0.001), obesity (68.4% vs. 13.2%, *p* < 0.001), diabetes (43.3% vs. 11.7%, *p* < 0.001), hypertension (76.4% vs. 28.2%, *p* < 0.001), hyperlipidemia (74.5% vs. 27.5%, *p* < 0.001), hypothyroidism (24.7% vs. 8.1%, *p* < 0.001), and cardiovascular disease (43.1% vs. 12.8%, *p* < 0.001).

### 3.2. Association Between OSA and Dry Eye Disease

As shown in Table 2, DED was significantly more common in the OSA group (19.4%) compared to controls (5.8%) (OR = 3.93, 95% CI: 3.82–4.04, *p* < 0.001). After adjusting for the comorbidities, OSA remained independently associated with DED (adjusted OR = 1.76; 95% CI: 1.70–1.82, *p* < 0.001). Subgroup analyses by race/ethnicity demonstrated that this association persisted across all evaluated populations—including Hispanic/Latino, non-Hispanic Asian, non-Hispanic Black, and non-Hispanic White participants (all *p* ≤ 0.001; Table 3). Adjusted odds ratios ranged from 1.68 to 2.13 across groups, indicating that the association between OSA and DED was consistent and robust across diverse demographic subgroups.

### 3.3. Association Between OSA and Meibomian Gland Dysfunction

The prevalence of MGD was higher in the OSA group (2.6%) compared to controls (1.0%) (OR = 2.68, 95% CI, 2.50–2.87; *p* < 0.0001). Following adjustment by comorbidities, the association remained statistically significant, with an adjusted OR of 1.43 (95% CI, 1.32–1.55; *p* < 0.0001) (Table 2). Subgroup analyses similarly showed that OSA was associated with higher odds of MGD across all racial/ethnic groups studied (all *p* ≤ 0.001; Table 3). Adjusted odds ratios ranged from 1.34 to 2.06, supporting a consistent relationship between OSA and MGD irrespective of racial and ethnic background.

## 4. Discussion

In this large, matched case–control cross-sectional study using the large AoURP database, we found a significant and independent association between OSA and both DED and MGD in a demographically diverse U.S. population. The 13% absolute difference in coded DED prevalence between individuals with OSA and matched controls indicates a notably higher documented burden of ocular surface disease among patients with OSA and may correspond to a meaningful increase in clinical demand at the population level. MGD demonstrated a smaller but still statistically significant association with OSA. These relationships persisted after adjustment for multiple cardiometabolic and lifestyle-related comorbidities, indicating that OSA was consistently associated with higher coded prevalence of both conditions independent of these factors. For clinicians caring for individuals with OSA, these findings support a heightened index of suspicion for ocular surface disease.

While this study corroborates prior evidence linking OSA with ocular surface disease, its primary contribution lies in validating these findings within a large, demographically diverse, U.S.-based cohort. Our findings are consistent with several recent smaller, single-center, or non-U.S. studies. For example, in a recent Taiwanese retrospective study of 86 patients with DED and 86 age-matched controls without DED, it was found that OSA was associated with both increased risk and severity of DED, and that CPAP use exacerbated dry eye symptoms in some patients [12]. Yu et al. conducted a community-based study involving 3070 adults in China, also found that sleep dysfunction assessed by Chinese version of Pittsburgh Sleep Quality Index score was significantly correlated with the severity of DED [15]. However, to our knowledge, our current study represents the largest and most demographically inclusive assessment of association between OSA and DED to date in the United States, made possible by the scale and diversity of the AoURP. The consistency of our findings across diverse cohorts within broader literature strengthens the associations, although directionality and causality cannot be inferred.

The pathophysiological mechanism for association between OSA and DED may be multifactorial. OSA is known to induce chronic intermittent hypoxia, oxidative stress, and systemic inflammation [3,18]. These same processes are implicated in the development and exacerbation of DED, in part through the upregulation of pro-inflammatory cytokines and matrix metalloproteinases on the ocular surface [7,19]. Additionally, autonomic nervous system dysregulation in OSA is associated with reduced lacrimal gland secretion and disruption of meibomian gland function, contributing to tear film instability and evaporative dry eye [20]. The association between OSA and meibomian gland dysfunction is well established, as highlighted by studies showing higher rates of gland dropout and altered meibum quality among patients with OSA [20]. Research specifically examining meibomian gland morphology revealed 20.1% gland loss in the upper eyelids of patients with OSA compared to 14.7% in controls, with corresponding lower eyelid losses of 19.0% versus 12.4% [20]. Patients with severe OSA exhibited significantly higher meibomian gland loss ratios compared to mild patients with OSA [21]. A prospective study of 103 patients with OSA undergoing uvulopalatopharyngoplasty surgery to treat OSA found that although meibomian gland structure remained unchanged at 3-month follow-up, tear film stability and ocular surface inflammation showed significant improvement [21]. Our findings add to this body of evidence, suggesting that meibomian gland dysfunction may partially serve as a mechanistic bridge between OSA and DED. Nonetheless, MGD is also commonly inconsistently coded across clinical settings, which may underestimate its true prevalence.

Although the AoURP did not collect data on the use of continuous positive airway pressure (CPAP), the first-line therapy for OSA, CPAP may play a role in the association between OSA and DED. Air leaks and increased ocular surface exposure, particularly with poorly fitted masks, can accelerate tear evaporation and worsen dry eye symptoms, thereby exacerbating DED [12,22,23]. Ophthalmologists and sleep medicine specialists should be aware of the heightened risk of DED in patients with OSA and consider routine ocular surface evaluation, especially for those experiencing ocular discomfort or using CPAP. An estimated 85% of individuals with clinically significant OSA are undiagnosed, due to the absence of noticeable symptoms, limited awareness of nighttime breathing disturbances, and inadequate screening practices [24]. The absence of CPAP usage data in AoURP represents a significant limitation. Because CPAP airflow and mask leakage can independently affect the ocular surface, the observed association between OSA and DED may partly reflect treatment effects. Nonetheless, comprehensive eye exams are seldom included in standard OSA care. Given the chronic nature of both OSA and DED, integrating DED evaluation into routine care for patients with OSA may help address an unmet need in this population. Strengthening partnerships between ophthalmologists and sleep medicine providers, in addition to educating patients about the ocular effects of CPAP therapy such as dry eye, may help prevent ocular health issues and enhance overall well-being in this at-risk group.

It is also notable that previous studies reported other ocular disorders related to DED have positive associations with OSA. Floppy eyelid syndrome (FES), although not studied in this study, is perhaps the most significant, with multiple studies indicating a markedly higher prevalence of FES among patients with OSA, likely due to the combination of mechanical eyelid trauma and systemic inflammation [25,26,27]. FES has also been reported to disrupt meibomian gland architecture and promotes inflammation through mechanical stress and matrix metalloproteinase-mediated tissue remodeling [28]. A study by Acar et al. found that patients with FES had low Schirmer and tear break-up time (TBUT) values, both commonly used to help diagnose DED, further supporting the significant association between FES and DED, and highlighting the interconnectedness of sleep disorders, eyelid pathology, and ocular surface disease [6,29]. Obesity is another common comorbidity, both as a risk factor for OSA and as an independent contributor to FES, possibly due to increased mechanical forces on the eyelids and systemic inflammation [30]. Although we cannot evaluate the association between OSA and FES due to the unavailability of FES diagnosis in the AoURP, these findings support the hypothesis that metabolic dysregulation and systemic inflammation, in conjunction with mechanical factors, may play a synergistic role in the pathogenesis of FES in patients with OSA. Glaucoma and ischemic optic neuropathy have also been linked to OSA, potentially via shared mechanisms of vascular dysregulation and hypoxic injury [31]. These converging lines of evidence suggest that the consequences of OSA for ocular health are far-reaching and appropriate treatment of OSA may result in better control of these symptoms.

Strengths of this study include the large sample size with subgroups, use of a geographically represented and diverse database, rigorous matching by demographics, and additional adjustment for confounders. Limitations include potential misclassification bias from EHR-based definitions for both OSA and DED, lack of information on OSA severity (mild, moderate, severe), OSA treatment status, family history of OSA, CPAP usage, or presence of floppy eyelid syndrome in the AoURP, and the cross-sectional nature of the analysis, which precludes assessment of temporality or causality. While SNOMED-CT coding provides standardized diagnostic classification, we could not validate the accuracy of the diagnosis of OSA and DED in the study due to misclassification of subtypes, inaccurate coding, or lack of specialist confirmation. The AoURP dataset lacked information on systemic medications known to affect tear film stability as well as hormone-related variables, the possibility of residual confounding cannot be excluded. Additionally, the ‘Other or Unknown’ racial/ethnic category (8.3% of the cohort) may introduce selection bias. Although such analyses were beyond the scope of this study, they represent an important direction for future work.

Future studies are needed to understand the molecular and cellular pathways linking intermittent hypoxia, inflammation, and ocular surface disease, to explore association between other sleep-disordered breathing syndromes and their ocular conditions, and to determine whether OSA treatment can mitigate ocular surface symptoms or improve DED outcomes. Early identification and management of DED may improve quality of life and visual outcomes in this high-risk population.

In conclusion, in this large, diverse U.S. cohort from the All-of-Us Research Program, OSA is independently associated with an increased coded prevalence of DED and MGD, even after accounting for demographic and clinical risk factors. These results underscore the need for integrated, multidisciplinary approaches to screening and managing ocular complications in patients with sleep-disordered breathing.

## Figures and Tables

**Table 1:** Characteristics of Participants with OSA and Matched Controls without OSA in the All of Us Research Program.

Characteristics of Participants at Enrollment	OSA (N = 59,804)	Matched Controls Without OSA (N = 179,412)	*p*
Age (years) *: Mean (SD)	61.95 (13.60)	61.95 (13.60)	1.00
Sex *: Female (%)	32,301 (54.0%)	96,903 (54.0%)	1.00
Race-Ethnicity *			1.00
Hispanic or Latino	7479 (12.5%)	22,437 (12.5%)	
Non-Hispanic Asian	848 (1.4%)	2544 (1.4%)	
Non-Hispanic Black	9288 (15.5%)	27,864 (15.5%)	
Non-Hispanic White	37,232 (62.3%)	111,696 (62.3%)	
Other or Unknown **	4957 (8.3%)	14,871 (8.3%)	
Current Smoking: Yes (%)	24,627 (13.7%)	6506 (10.9%)	<0.001
Obesity: Yes (%)	40,881 (68.4%)	23,653 (13.2%)	<0.001
Diabetes: Yes (%)	25,866 (43.3%)	20,958 (11.7%)	<0.001
Hypertension: Yes (%)	45,678 (76.4%)	50,570 (28.2%)	<0.001
Hyperlipidemia: Yes (%)	44,539 (74.5%)	49,294 (27.5%)	<0.001
Hypothyroidism: Yes (%)	14,749 (24.7%)	14,480 (8.1%)	<0.001
Cardiovascular Disease: Yes (%)	25,785 (43.1%)	23,047 (12.8%)	<0.001

* Cases and controls were exactly matched by age, sex and race/ethnicity. ** Other or unknown included those who had more than one race category, those who had other race not listed, and those with race information unknown. Abbreviations: SD = Standard deviation; OSA = obstructive sleep apnea.

**Table 2:** Univariate Analysis and Multivariate Analysis for Association between OSA and Dry Eye Disease and Meibomian Gland Dysfunction in the All of Us Research Program.

	Dry Eye Disease			Meibomian Gland Dysfunction		
	Controls (N = 179,412)	OSA (N = 59,804)	*p*	Controls (N = 179,412)	OSA (N = 59,804)	*p*
Prevalence rate (%)	10,387 (5.8%)	11,626 (19.4%)		1778 (1.0%)	1561 (2.6%)	
Unadjusted OR (95% CI)	1.00	3.93 (3.82, 4.04)	<0.001	1.00	2.68 (2.50, 2.87)	<0.001
Adjusted OR (95% CI) *	1.00	1.76 (1.70, 1.82)	<0.001	1.00	1.43 (1.32, 1.55)	<0.001

Abbreviations: OR = odds ratio; 95% CI = 95% confidence interval; OSA = obstructive sleep apnea. * Adjusted by smoking status, obesity, diabetes, hypertension, hyperlipidemia, cardiovascular disease, hypothyroidism.

**Table 3:** Univariate Analysis and Multivariate Analysis for Association between OSA and Dry Eye Disease and Meibomian Gland Dysfunction stratified by Race-Ethnicity Groups.

		Dry Eye Disease			Meibomian Gland Dysfunction		
Hispanic or Latino		**Controls (N = 22,437)**	**OSA** **(N = 7479)**	***p***	**Controls (N = 22,437)**	**OSA** **(N = 7479)**	***p***
	Prevalence rate (%)	1450 (6.5%)	1777 (23.8%)		231 (1.0%)	275 (3.7%)	
	Unadjusted OR (95% CI)	1.00	4.51 (4.18, 4.86)	<0.001	1.00	3.67 (3.07, 4.38)	<0.001
	Adjusted OR (95% CI) *	1.00	2.13 (1.95, 2.33)	<0.001	1.00	2.06 (1.67, 2.53)	<0.001
Non-Hispanic Asian		**Controls (N = 2544)**	**OSA** **(N = 848)**	***p***	**Controls (N= 2544)**	**OSA** **(N = 848)**	***p***
	Prevalence rate (%)	162 (6.4%)	195 (23.0%)		34 (1.3%)	39 (4.6%)	
	Unadjusted OR (95% CI)	1.00	4.39 (3.50, 5.50)	<0.001	1.00	3.56 (2.23, 5.68)	<0.001
	Adjusted OR (95% CI) *	1.00	1.93 (1.49, 2.50)	<0.001	1.00	1.70 (1.01, 2.86)	<0.001
Non-Hispanic Black		**Controls (N = 27,864)**	**OSA** **(N = 9288)**	***p***	**Controls (N = 27,864)**	**OSA** **(N = 9288)**	***p***
	Prevalence rate (%)	1579 (5.7%)	2065 (22.2%)		255 (0.9%)	270 (2.9%)	
	Unadjusted OR (95% CI)	1.00	4.76 (4.44, 5.11)	<0.001	1.00	3.24 (2.73, 3.85)	<0.001
	Adjusted OR (95% CI) *	1.00	1.74 (1.59, 1.89)	<0.001	1.00	1.41 (1.15, 1.73)	0.001
Non-Hispanic White		**Controls (N = 111,696)**	**OSA** **(N = 37,232)**	***p***	**Controls (N = 111,696)**	**OSA** **(N = 37,232)**	***p***
	Prevalence rate (%)	6352 (5.7%)	6551 (17.6%)		1120 (1.0%)	861 (2.3%)	
	Unadjusted OR (95% CI)	1.00	3.54 (3.41, 3.67)	<0.001	1.00	2.34 (2.14, 2.56)	<0.001
	Adjusted OR (95% CI) *	1.00	1.68 (1.61, 1.76)	<0.001	1.00	1.34 (1.21, 1.49)	<0.001

Abbreviations: OR = odds ratio; 95% CI = 95% confidence interval; OSA = obstructive sleep apnea. * Adjusted by smoking status, obesity, diabetes, hypertension, hyperlipidemia, cardiovascular disease, hypothyroidism.

## Data Availability

The data that support the findings of this study are available from the corresponding author, GSY, upon request.

## Abbreviations

The following abbreviations are used in this manuscript:

AoURP	All-of-Us Research Program
CI	Confidence Interval
CPAP	Continuous Positive Airway Pressure
DED	Dry Eye Disease
EHR	Electronic Health Record
FES	Floppy Eyelid Syndrome
IRB	Institutional Review Board
MGD	Meibomian Gland Dysfunction
OR	Odds Ratio
OSA	Obstructive Sleep Apnea
*p*	Probability value
SD	Standard Deviation
SNOMED-CT	Systematized Nomenclature of Medicine–Clinical Terms
TBUT	Tear Break-Up Time
U.S.	United States
